# Long-term health and germline transmission in transgenic cattle following transposon-mediated gene transfer

**DOI:** 10.1186/s12864-018-4760-4

**Published:** 2018-05-23

**Authors:** Soo-Young Yum, Song-Jeon Lee, Sin-Gi Park, In-Gang Shin, Sang-Eun Hahn, Woo-Jae Choi, Hee-Soo Kim, Hyeong-Jong Kim, Seong-Hun Bae, Je-Hyeong Lee, Joo-Yeong Moon, Woo-Sung Lee, Ji-Hyun Lee, Choong-Il Lee, Seong-Jin Kim, Goo Jang

**Affiliations:** 1 Department of Theriogenology, College of Veterinary Medicine and the Research Institute of Veterinary Science, Seoul National University, #631 Building 85, Gwanak-ro, Gwanak-gu, Seoul, 08826 Republic of Korea; 2 Embryo Research Center, Seoul Milk Coop, Gyeonggi-do, 12528 Republic of Korea; 3 Bioinformatics Team, Theragen Etex Bio Institute, Advanced Institutes of Convergence Technology, Kwanggyo Technovalley, Suwon, 16229 Republic of Korea; 4 Emergence Center for Food-Medicine Personalized Therapy System, Advanced Institutes of Convergence Technology, Seoul National University, Gyeonggi-do, 16229 Republic of Korea

**Keywords:** Germline transmission, Next-generation sequencing, *Sleeping beauty*, *PiggyBac*, Transgenic cattle

## Abstract

**Background:**

Transposon-mediated, non-viral gene delivery is a powerful tool for generating stable cell lines and transgenic animals. However, as multi-copy insertion is the preferred integration pattern, there is the potential for uncontrolled changes in endogenous gene expression and detrimental effects in cells or animals. Our group has previously reported on the generation of several transgenic cattle by using microinjection of the Sleeping Beauty (SB) and PiggyBac (PB) transposons and seeks to explore the long-term effects of this technology on cattle.

**Results:**

Transgenic cattle, one female (SNU-SB-1) and one male (SNU-PB-1), reached over 36 months of age with no significant health issues and normal blood parameters. The detection of transgene integration and fluorescent signal in oocytes and sperm suggested the capacity for germline transmission in both of the founder animals. After natural breeding, the founder transgenic cow delivered a male calf and secreted milk containing fluorescent transgenic proteins. The calf expressed green fluorescent protein in primary cells from ear skin, with no significant change in overall genomic stability and blood parameters. Three sites of transgene integration were identified by next-generation sequencing of the calf’s genome.

**Conclusions:**

Overall, these data demonstrate that transposon-mediated transgenesis can be applied to cattle without being detrimental to their long-term genomic stability or general health. We further suggest that this technology may be usefully applied in other fields, such as the generation of transgenic animal models.

**Electronic supplementary material:**

The online version of this article (10.1186/s12864-018-4760-4) contains supplementary material, which is available to authorized users.

## Background

Transposon-mediated gene delivery is a valuable technique for use in gene therapy and ex vivo gene delivery and for in vitro cell line and animal model generations [1–4]. Of the several DNA transposons available, Sleeping Beauty (SB) and PiggyBac (PB) transposons have been the most widely used to deliver exogenous genes into cell lines and to generate transgenic animals. In mice and rats, it is well established that transposon-mediated transgenesis can result in germline transmission to create transgenic offspring. Transposons such as SB and PB have also been used successfully to produce transgenic pigs, sheep, goats and cattle [5–10], and in the case of the transgenic pigs, germline transmission was also observed [11].

The transposon system is particularly valuable as it mediates both the efficient genomic integration and stable expression of transgenes in target cells or animals. The transposase enzyme binds specifically to the recognition sequences of the transposon and induces the ‘cut-and-paste’ integration of a transposon vector into the genome in a site-specific manner (SB integrates at TA sequences and PB integrates at TTAA) free from vector backbone DNA [12]. This system can be used in conjunction with various non-viral delivery systems, such as chemical-based transfection, electroporation or microinjection. In comparison to viral delivery and transgene integration systems, transposon systems are more secure and safe to use [13, 14] and this has led to NIH-OBA and FDA approval for testing of the SB transposon in humans [15–17].

In vitro studies have shown that the random nature of SB and PB insertion events can lead to insertional mutagenesis [4, 18]. However, as transposon-mediated gene transfers can also integrate within non-coding regions, the system is still considered far safer than viral delivery systems in terms of potential geno-toxicity. In support of this, our study on transgene integration sites in transgenic cattle demonstrated that all transgenes were integrated within non-coding and non-functional regions [5]. However, such genomic analyses may not be entirely predictive of general health in transgenic animals. Although transgenic cattle have been generated via transposon-, viral vector- or somatic cell nuclear transfer-mediated gene transfers, to date there has been no report on long-term monitoring of health issues in such cattle or their offspring. Therefore, we have sought to test our hypothesis that multi-copy transgene integration in transgenic cattle (founders) will not affect their long-term survival (over 3 years) and, further, will undergo germline transmission.

## Methods

### Animals

All the transgenic cattle involved in this study were reported in our previous publication [5]. Briefly, the transgenic cattle (females SNU-SB-1 and SNU-PB-2, and male SNU-PB-1) were derived from embryos which had undergone microinjection in order to introduce either of the two transposon systems (SB or PB) as indicated.

### Cell isolation and culture

Primary cultures of fibroblasts were derived from ear skin biopsies of transgenic cattle. The ear skin was cut into pieces of 1–4 mm in size with a sterile scalpel before being washed several times and incubated at 37 °C for 16 h in HBSS supplemented with collagenase (Collagenase type IV, Gibco). The dispersed cells were washed in HBSS and cultured in DMEM supplemented with antibiotics and 10% fetal calf serum.

### Semen collection and freezing

Semen from the male transgenic founder was collected using an artificial vagina (Fujihira Industry, Tokyo, Japan) containing warm water at 50–55 °C. The collected semen was immediately transported to the laboratory for freezing. The semen was diluted 1:1 with OPTIXcell (IVM technologies, France) and incubated at room temperature for 10 min. The semen was further diluted 1:1 to a sperm concentration of around 5.0 × 10^7^ cells/mL before incubation at 4 °C for 2 h. The concentrated sperm solution was loaded into a 500 μL semen straw (IMV technologies, France) and sealed with straw powder (Fujihira Industry, Tokyo, Japan). The straw underwent freezing 5.0 cm above the surface of liquid nitrogen for 30 min and was then plunged into a liquid nitrogen tank for storage.

### In vitro oocyte maturation, fertilization and culture of embryos

Ovaries were obtained from a local abattoir and maintained in saline at 35 °C during transport to the laboratory. Cumulus–oocyte complexes (COCs) from follicles 2 to 8 mm in diameter were aspirated using an 18-guage needle, selected and collected in a conical tube. The sediment was washed three times with HEPES-buffered tissue culture medium-199 (TCM-199; Invitrogen, Carlsbad, CA, USA) supplemented with 2 mM NaHCO_3_ (Sigma–Aldrich Corp., St. Louis, MO, USA), 10% FBS and 1% penicillin–streptomycin (*v*/v). For in vitro maturation, COCs were cultured for 22 h in 450 μL TCM-199 supplemented with 0.005 AU/mL FSH (Sigma–Aldrich), 10% FBS, 1 μg/mL 17β-estradiol (Sigma–Aldrich) and 100 μM Cysteamine (Sigma-Aldrich) at 39 °C under 5% CO_2_.

The Percoll gradient method for the separation and purification of motile spermatozoa has been described in detail elsewhere [19]. Briefly, spermatozoa were purified from thawed semen straws by density-gradient centrifugation on a Percoll discontinuous gradient (45–90%) at 1500 rpm for 15 min. The Percoll density gradient was prepared by layering 1 mL of 45% Percoll solution onto 1 mL of 90% Percoll solution in a 15 mL conical tube. The thawed semen was layered onto the top of the Percoll gradient solution and the tube was centrifuged. The pellet was washed twice with TALP by centrifugation for 5 min at 1500 rpm. The active, motile spermatozoa from the pellet were added to droplets containing matured oocytes. Oocytes were inseminated on day 0 with 1–2 × 10^6^ spermatozoa/mL for 18 h in IVF-TALP medium (Nutricell) under mineral oil. The fertilized oocytes were denuded and cultured in two-step defined culture medium (5 days in D1 medium before transfer to D2 medium) at 39 °C in an atmosphere of 5% O_2_, 5% CO_2_ and 90% N_2_ [20].

### Blood analysis and veterinary care

A veterinarian collected 5 ml whole blood samples from the jugular vein for blood analysis and monitored regularly general health condition. Some were used for Complete Blood Count (CBC) (Hemavet 950, Drew Scientific, USA) and the others were used for serum chemistry analysis (BS-400, Mindray, China).

One transgenic cow (SNU-PB-2) was injured by other cattle, leading to severe respiratory distress. Under the advice of veterinarians, we decided to euthanize the cow. The planned method of euthanasia was administration of a general anesthetic reagent before the use of pentobarbital. However, due to severe organ damage caused by the respiratory distress, only the general anesthetic (Xylazine, BAYER; 0.15 mg/kg intravenous) was administered to be euthanasia.

### Library preparation for massively parallel sequencing

Purified genomic DNA from F0 (SNU-SB-1, SNU-PB-1) samples and the SNU-F1–1 sample was randomly sheared using a Covaris S2 Ultrasonicator to yield DNA fragments of on average 350 bp in size. Library preparation was performed using the Illumina TruSeq DNA PCR-free preparation kit. Adaptor enrichments were performed using PCR according to the manufacturer’s instructions. The final library size and quality were evaluated by electrophoresis using an Agilent High Sensitivity DNA kit. The 150 bp paired-end reads were sequenced with an Illumina HiSeq 4000 platform. Further image analysis and base calling were performed with RTA 2.7.3 (Real Time Analysis) and bcl2fastq v2.17.1.14.

### Read alignment

Sequenced reads were filtered using sickle (v1.33) with a Phred quality threshold of 20 to derive high-quality reads. The remaining reads were mapped against the *Bos taurus* genome (UMD 3.1, http://asia.ensembl.org/Bos_taurus/Info/Annotation) and the transgene sequence simultaneously using BWA ver. 0.7.5a. After mapping, duplicates were marked using Picard ver. 1.128 and local realignment was then performed using GATK ver. 3.6–0.

### Variant analysis

Multi-sample calling (including F0 samples) of single nucleotide variants (SNV) and insertions and deletions (INDELs) was performed using GATK ver.3.6–0 with UnifiedGenotyper. After multi-sample calling, variants were filtered using a genotype quality value cutoff of 60. The SnpEff software was used together with the UMD3.1.79 *Bos taurus* Ensembl annotation set to predict the functional effects of the variants detected.

### Assessment of genomic stability

In order to investigate potential genomic instability, we classified SNPs and INDELs into three groups: RefHom (homozygous reference genotype), Hetero (heterozygous genotype) and AltHom (homozygous alteration genotype). Genetic variants (SNPs and INDELs) were assigned using the following criteria: (1) if the sequence is classified as RefHom, the ratio to the reference allele depth was more than 90%; (2) if the sequence is classified as Hetero, the ratio to the reference allele depth was more than 40% and less than 60%; (3) if the sequence is classified as AltHom, the ratio to the altered allele depth was more than 90%. We also removed the mitochondrial genome, X chromosome and unanchored scaffolds from further analysis. Finally, we searched remaining variants as de novo mutations rather than inherited sequences.

### Identification of copy number variations (CNVs)

The Control-FREEC software was applied for the identification of copy number changes in the genomes of the transgenic cattle. The software is used for calculation of the ploidy of regions of interest, with the copy number value calculated for a 50 kb window in the region of interest, following GC content read count normalization, and compared to a normal autosomal ploidy value of two.

### Detection of transgene insertion sites

Transgene insertion sites were identified some soft-clipped nucleotide following mapping with BWA. Some soft-clipped nucleotides could be determined by a Smith-Waterman-like scoring scheme in the BWA software. The candidate insertion sites were inferred by inspection of the mapped pattern in the soft-clipped sequences. Delly software was also applied in parallel for estimation of genomic structural variation as an indicator of transgene insertion. Finally, we manually inspected the candidate sites with IGV software.

### Calculation of telomere length using the whole genome sequence

Reads that were rich in telomere sequences were extracted from the whole genome sequence dataset and their relative length determined. We then applied TelSeq software to reveal any difference in the number of copies of TTAGGG between transgenic and control genomes through calculation of the frequency of reads.

### Measurement of fluorescence intensity

To quantify the fluorescence intensities of samples from SNU-F1–1 and SNU-F1–2, images of cells of the same passage and density were acquired. Using ImageJ (v1.50, NIH), an equally-sized region was selected using the square-drawing function of the drawing/selection tools and pixel measurements (area, mean gray value and integrated density) acquired from this region of interest. The integrated density for each cell was calculated for the region of interest.

## Results

### Health monitoring using blood analysis

**Fig. 1:**
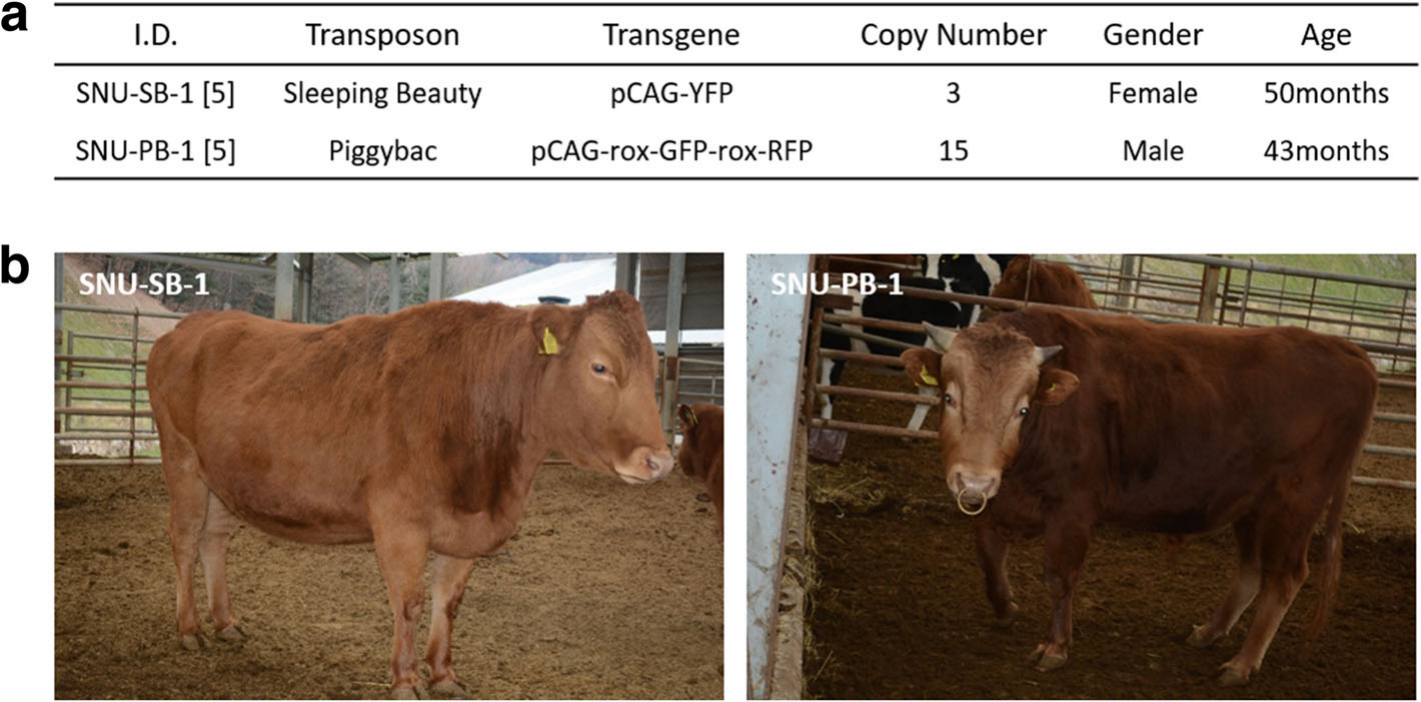
Overview of transposon-derived transgenic cattle in this study. **a** Summary of transgene and general information for the transgenic cattle. **b** Recent images of the transgenic cattle. Left: SNU-SB-1; right: SNU-PB-1

**Fig. 2:**
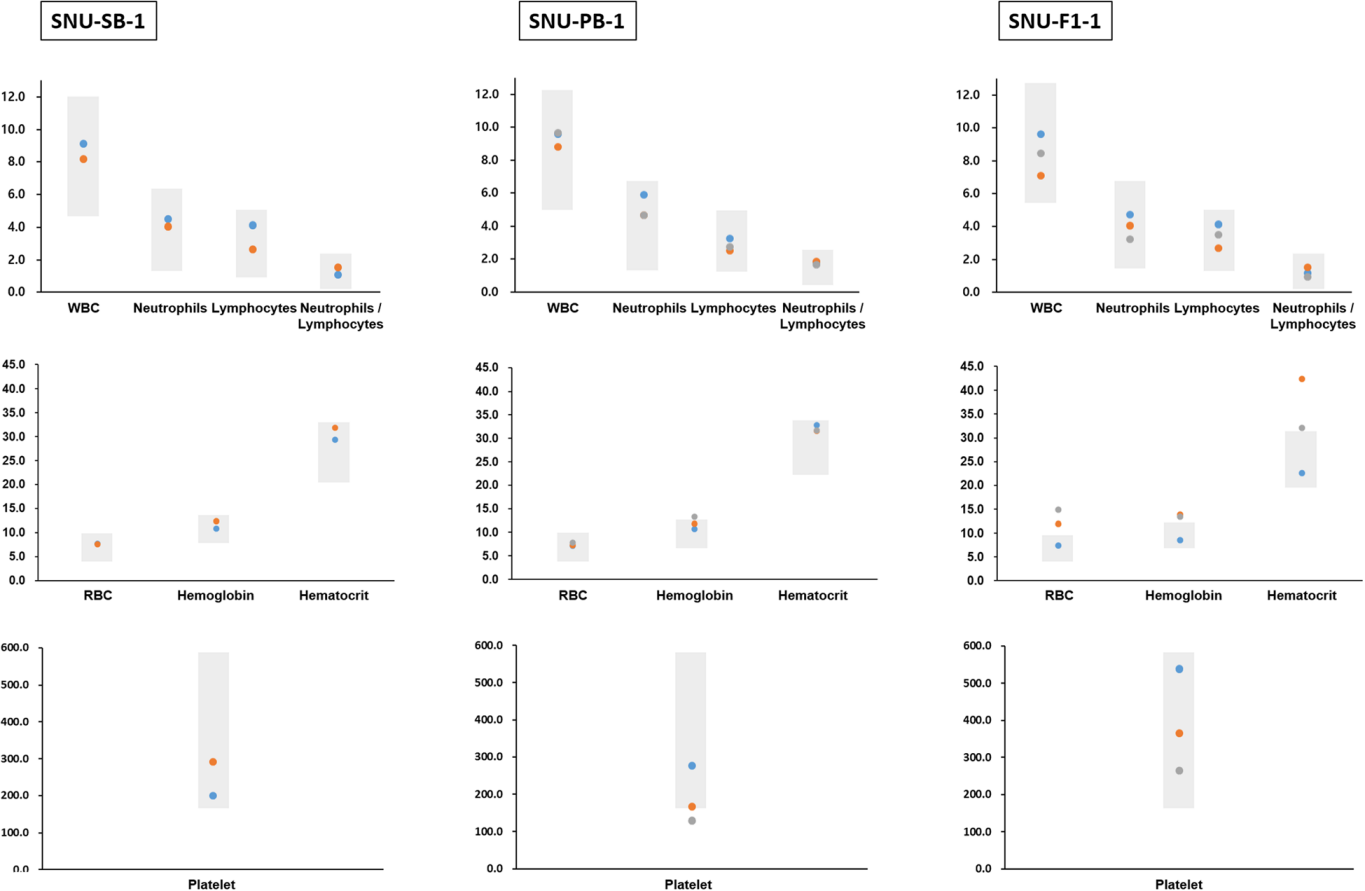
Analysis of blood parameters from three transgenic cattle (SNU-SB-1, SNU-PB-1 and SNU-F1–1). Samples were collected three times at different ages. One sample for counting of WBC from SNU-SB-1 failed due to blood coagulation, but chemical analysis of the serum was performed as planned (see Additional file 1). Circles indicate dates of blood collection and analysis; Orange circle: 26/08/2016; Blue circle: 26/10/2016; Gray circle: 27/03/2017; WBC: White blood cells; RBC: Red blood cells; Gray box: reference range

Both female (SNU-SB-1) and male (SNU-PB-1) transgenic cattle reached ages of 50 and 43 months, respectively, without any health problems (Fig. 1). Analysis of blood parameters (white blood cells, WBC; red blood cells, RBC; platelets) showed no significant difference between the blood of the transgenic animals and that of the reference (Fig. 2). Furthermore, there was no significant difference in various serum chemical parameters, including alanine transaminase (ALT), aspartate transaminase (AST), and blood urea nitrogen (BUN), between the transgenic animals and the reference (Additional file 1).

### Germline transmission of the transgene

**Fig. 3:**
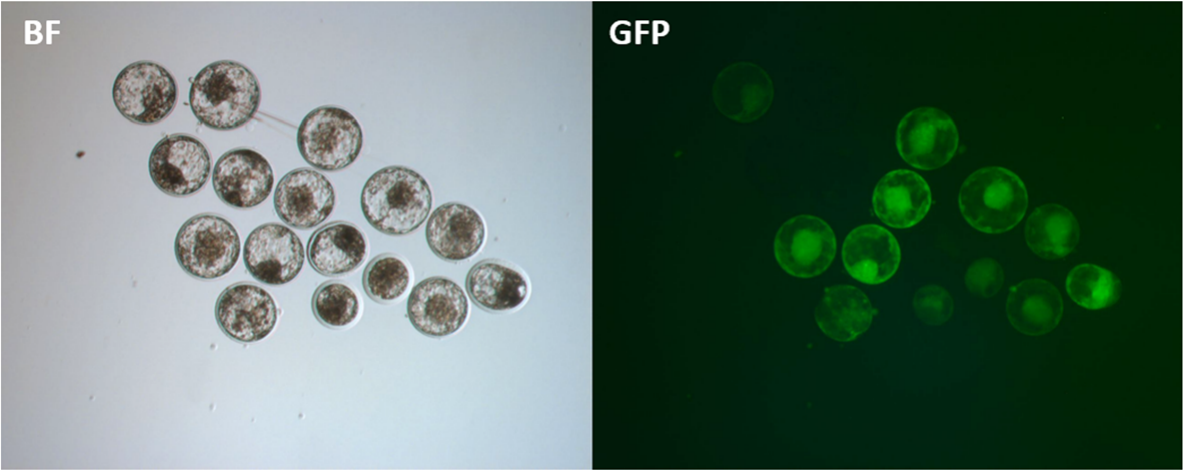
Germline transmission of transgenes in spermatozoa from SNU-PB-1. Blastocyst stage embryos express GFP after IVF using frozen-thawed semen from SNU-PB-1. BF: bright field; GFP: GFP field

To assess the germline transmission in sperm of transgenes introduced by transposons, sperm was harvested from SNU-PB-1 after puberty using manual ejaculation and cryopreserved to create frozen semen stocks. Over 200 straws were produced and preserved in liquid nitrogen for future use. The motility of frozen-thawed semen was normal, and the semen was used for in vitro fertilization (IVF) together with wild-type oocytes. Around 88% of blastocysts expressed green fluorescent protein (GFP) in every round of IVF undertaken (Fig. 3). Thus, the frozen sperm can be used to rapidly increase the population of transgenic cattle. These results demonstrate that germline transmission of transgenes introduced by transposon systems is possible.

### Natural birth of an F1 calf from transgenic cattle

**Fig. 4:**
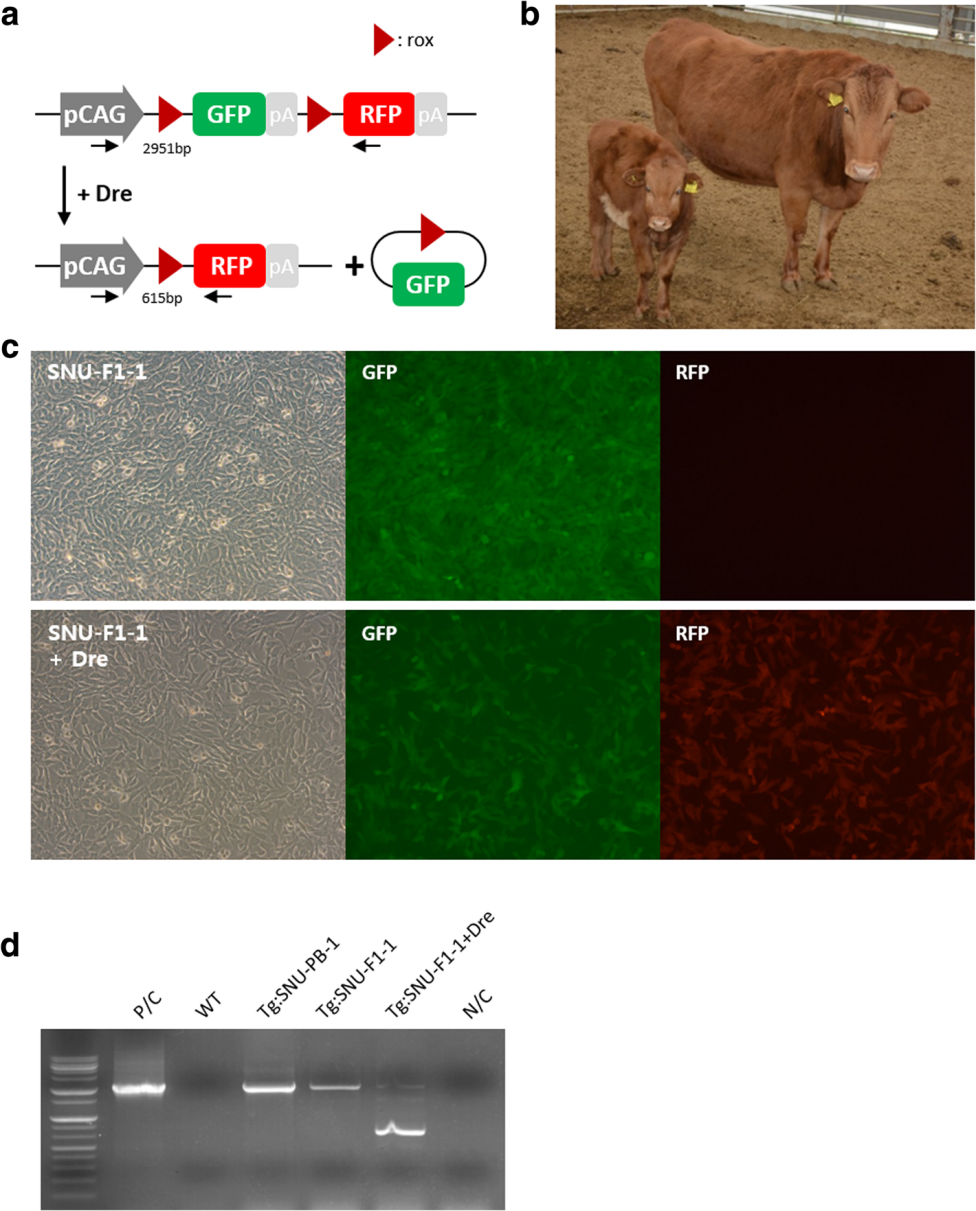
Detection of transgene expression in SNU-F1–1. **a** Schematic of Dre-rox recombination in constructs used in this study. **b** Image of SNU-F1–1 (left) and its mother, SNU-SB-1 (right). **c** Primary cells from the ear skin of SNU-F1–1 express the transgenic reporter protein, GFP (upper). RFP expression is detected following transfection of ear skin cells with Dre recombinase (lower). **d** PCR analysis of Dre-rox recombination using genomic DNA from the cells derived from SNU-F1–1. P/C, positive control (PB-CA-Rox-GFP-Rox-RFP vector); WT, genomic DNA from wild type cattle; Tg:SNU-PB-1, genomic DNA from the blood of SNU-PB-1; Tg:SNU-F1–1, genomic DNA from the blood of SNU-F1–1; Tg:SNU-F1–1 + Dre, genomic DNA from cells from SNU-F1–1 which have undergone transfection with Dre recombinase; N/C, negative control (nuclease-free water)

SNU-SB-1 was mated naturally with SNU-PB-1 in order to assess the stability of germline transmission of the transgene to the resultant offspring. The F1 (SNU-F1–1, male) was delivered without any assistance following the gestation period (Fig. 4b) and a physical examination concluded that there were no congenital defects. Expression of the GFP transgene in the eyes of the calf was apparent without the use of equipment. To further investigate the germline transmission of the transgene, skin fibroblasts were isolated from SNU-F1–1, cultured and expanded for genomic analysis. All of the fibroblasts homogenously expressed GFP (Fig. 4c) and PCR analysis of the genomic DNA from these cells demonstrated the presence of GFP transgenes in the genome (Fig. 4d). The construct integrated into the genome of the SNU-PB-1 father contained rox-flanked (froxed) GFP followed by red fluorescent protein (RFP) [5]. We therefore expected that if the same parental transgenes had been successfully transmitted to SNU-F1–1, the expression of Dre recombinase should excise the froxed GFP from the genome, leaving only a single rox site and RFP in the genome (Fig. 4a). As expected, electroporation of Dre recombinase into cells from SNU-F1–1 was sufficient to induce the expression of RFP and the deletion of froxed GFP was confirmed by PCR analysis (Fig. 4c–d). In contrast, we did not detect transmission of yellow fluorescent protein (YFP) transgenes from the mother (Additional file 2: Figure S1). These data indicate the successful transmission of transgenes from SNU-PB-1 to F1 offspring. Furthermore, the F1 calf SNU-F1–1 was healthy, with no significant abnormalities detected by blood analysis (Fig. 2).

We examined wild-type milk and transgenic milk by confocal microscopy to determine whether the transgenic fluorescent protein would be detected in milk from SNU-SB-1 after delivery of SNU-F1–1. As expected, YFP was observed in the milk from SNU-SB-1 (Additional file 3: Figure S2). This result suggests that transposon-derived transgenic cattle can be used as bioreactors for the production of recombinant proteins.

### Whole genome sequencing to determine integration sites and genomic stability

**Fig. 5:**
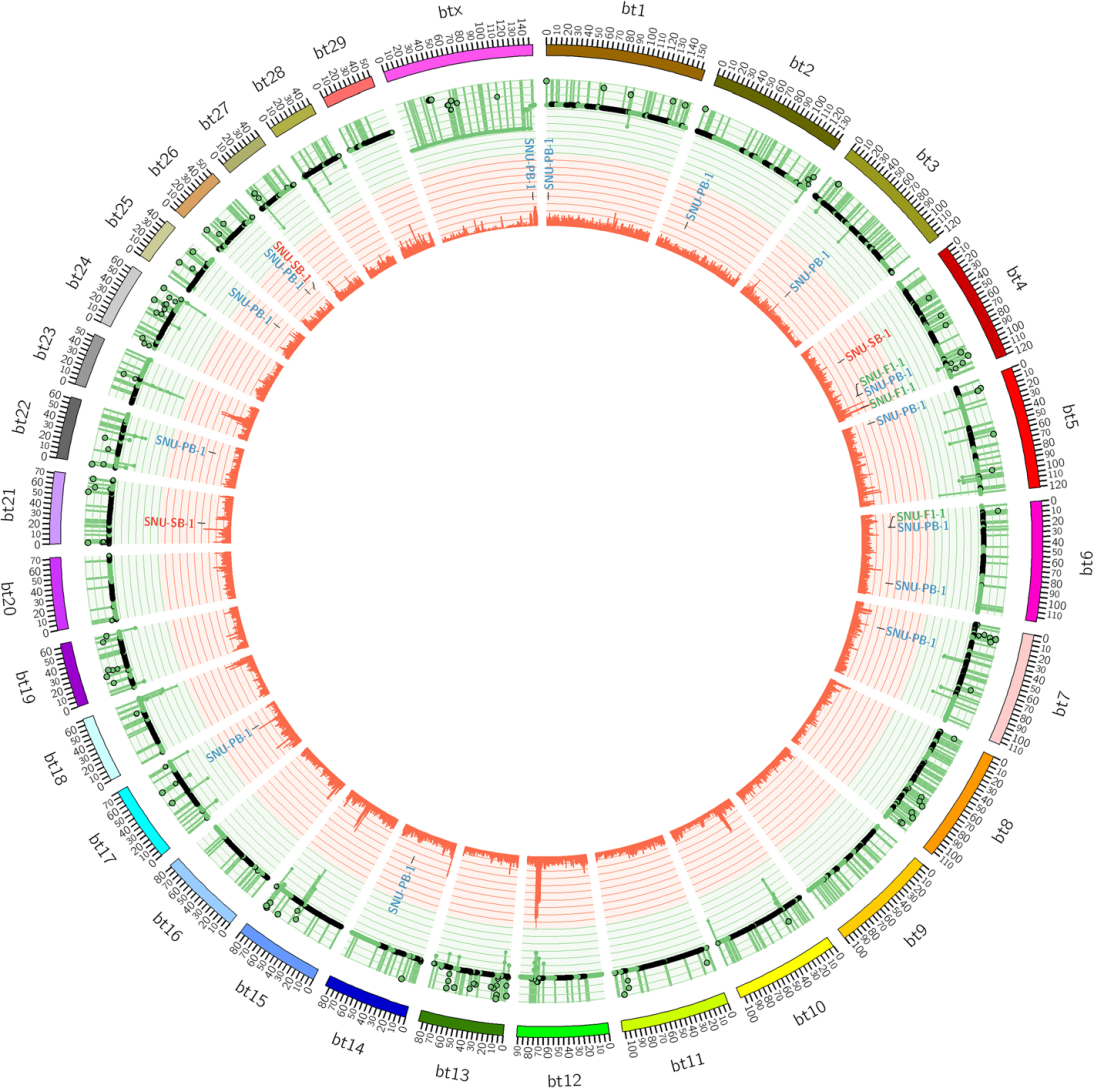
Overview of genomic variation in SNU-F1–1. Reference chromosomes from bt1 to btX are denoted by colored boxes at the outer edge. Plots denoting copy number variation (CNV; black dot plots in the green area), coverage (green line plot in the green area) and SNP density (orange histogram in orange area) for the SNU-F1–1 genome are shown for each 10 kb window

To confirm the integration sites of the transgenes within the maternal and paternal genomes, whole genome sequencing was performed with next-generation sequencing (NGS). NGS has been widely used for high-throughput genomic analysis, such as molecular characterization and structure variation. We confirmed the transgene insertion sites previously identified in the F0 transgenic cattle [5, 21]. NGS of the SNU-F1–1 genome was performed to determine the transgene insertion sites. The sequencing showed that a total of three copies of the paternal transgene were transmitted to the F1 calf, and we determined the sites of integration for the transgenes to be in non-coding regions (Table 1). Transgene insertion sites were verified by 5′ junction sequence analysis using a specific primer set that anneals to the unique genome-to-transposon junction in chromosomes 4 (two sites) and 6 (Additional file 4: Figure S3). Additionally, we compared the genome of SNU-F1–1 to those of its parents (F0 samples) and identified 147 heterozygous de novo mutations and 2 homozygous de novo mutations differing from the maternal and paternal genomes (Table 2). The heterozygous de novo mutations were classified by position into intergenic (125, 78.61%), intronic (33, 20.76%) and exonic (1, 0.63%, in the ENSBTAG00000038261 gene) mutations, with most occurring in the intergenic and intronic regions. In addition, the homozygous de novo mutations were identified mistaken searched variants because they occurred in the long terminal repeat (LTR) region. The number of de novo mutations is consistent with previous studies [22], corresponding to a mutation rate of 5.62 × 10^− 8^ per position per generation per genome. And then, we identified the telomere length is 10.23. We have summarized the SNVs, INDELs and CNVs detected (Fig. 5).

**Table 1:** All integration sites in SNU-F1–1

No.	Chromosome	Insertion site	Orientation	5′ gene	3′ gene
1	4	95,433,564–95,434,563	Forward	TSGA13	MKLN1
2	4	113,823,097–113,823,101	Forward	ENSBTAG00000001198.5	ENSBTAG00000046257.1
3	6	20,085,913–20,086,912	Forward	DKK2	GIMD1

**Table 2:**
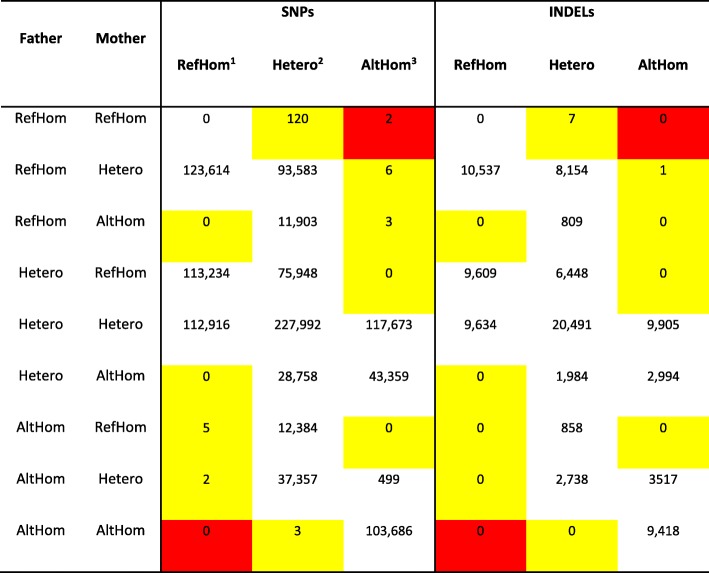
Pattern of SNPs and INDELs from parental DNA

^1^Homozygous reference genotype

^2^Heterozygous genotype

^3^Homozygous altered genotype

Yellow box: heterozygous de novo mutation not detected in parental DNA

Red box: homozygous de novo mutation not detected in parental DNA

### Correlation of GFP expression level with transgene copy number

**Fig. 6:**
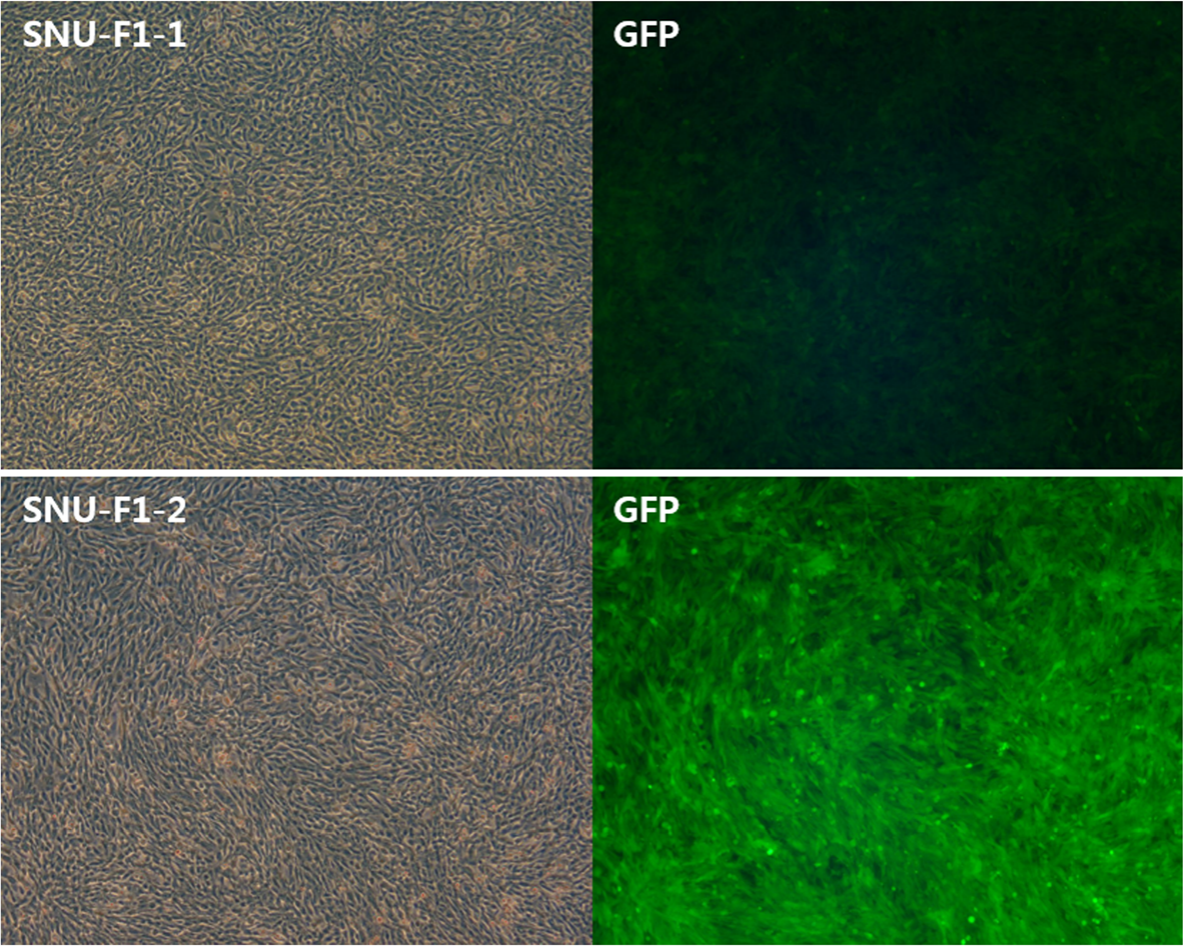
Expression level of GFP in cells from SNU-F1–1 and SNU-F1–2. Brightfield and fluorescent images of cells from SNU-F1–1 (upper) and SNU-F1–2 (lower). GFP: GFP field

There are several studies which demonstrate a correlation between the level of protein expression and transgene copy number [23, 24]. To investigate the relationship between GFP expression level and transgene copy number, we analyzed the skin fibroblasts from SNU-F1–1 and fetal fibroblasts derived from the fetus (SNU-F1–2) of another pregnant transgenic animal, SNU-PB-2 [5]. SNU-PB-2 became pregnant but was gravely injured by other cattle and had to be euthanized. Fluorescent signal was confirmed in the recovered uterus, ovary and oocytes of SNU-PB-2 (Additional file 5: Figure S4). NGS analysis of the genome of SNU-F1–2 identified six transgene integration sites and no genomic instability (Additional file 6: Table S1 and Additional file 7: Figure S5). Both SNU-F1–1 and SNU-F1–2 were generated from a single embryo and there was no mosaicism. As expected, cells from SNU-F1–2 (six copies) showed an approximately 2.2-fold higher expression level of GFP compared to cells from SNU-F1–1 (three copies) (Fig. 6). This result confirms previous work showing that transgene copy number is an important factor to determine the level of transgenic protein expression in transgenic cattle.

## Discussion

Transgenesis in cattle shows great promise for providing insights into basic embryogenesis and disease mechanisms (through the creation of disease models), as well as clear potential for protein production and isolation through using cattle as bioreactors. Thus it has long been of interest as a biotechnology in agricultural and veterinary science. However, its progress has been hampered at the practical level by a low efficiency of gene delivery, abnormal reprogramming in cloned embryos and a low success rate for obtaining cloned transgenic offspring with frequent complications (i.e., early embryonic loss and sudden death) [25, 26].

Transposon systems have been extensively applied when generating transgenic animal models and have been successfully utilized in zebrafish [27], mice [28], rats [29], pigs [30] and cattle [7]. Moreover, previous studies have verified germline transmission when using transposons in transgenic zebrafish [31], rodents [32, 33] and pigs [6, 11]. As cattle have a long gestational term and time to puberty, there has only been one previous study, which used lentivirus-mediated transgenesis, to investigate germline transmission in transgenic cattle [34]. In the present study, we have demonstrated that both female and male transgenic cattle with multi-copy integration of transposon-derived transgenes can reach maturity without health issues and we have verified germline transmission through the inheritance of their transgenes by the F1 generation.

Due to the cut-and-paste action of DNA transposons, it has long been presumed that transposon-mediated transgenesis could create additional genomic instability [1, 35]. However, many studies have demonstrated that transposons can be used safely and without inducing genomic instability [5, 6, 29, 36–38]. Additionally, in our previous report, all transgenes inserted using either SB or PB in multiple cattle were found to be integrated into non-coding regions. Consistent with previous reports [38], there was no significant difference between the SNU-F1–1 offspring and its parents in terms of SNP, CNV, structural variation (SV) or telomere length as assessed by NGS analysis (Fig. 5). Transgene integration sites were identified in three loci in SNU-F1–1 via NGS analysis (Table 1), and were determined to be only from the paternal genome (SNU-PB-1). As the transgenes integrated into the maternal genome (SNU-SB-1) were heterozygous, it is possible that, following haploid cell generation, an oocyte lacking transgenes was fertilized by the sperm containing the GFP transgenes. As in our previous report, we found that the transgenes in the offspring (SNU-F1–1) were integrated into sites bearing the PB preferred integration sequence, “TTAA” (Additional file 4: Figure S3) [5]. Two integration sites were identical to those seen in SNU-PB-1. One site was not identified in our previous report investigating integration sites in the paternal genome, possibly because that analysis was performed on material from the blood [5] and did not assess integration sites in other organs, such as the testes.

This report also demonstrates that transgenic cattle can act as potential bioreactors for the secretion of exogenous proteins in milk [39–41], as we identified the production of YFP in milk from SNU-SB-1 (Additional file 3: Figure S2). Further, SNU-F1–1 was fed the YFP-containing milk from SNU-SB-1 for 7 months until weaning. We note that even though the calf consumed milk containing fluorescent proteins for a long period, it has not shown any health issues to date, and so conclude that consumption of transgenic fluorescent protein does not affect health.

The position of the transgene integration site within the genome determines its level of expression or silencing, an effect illustrated by the mosaicism seen during germline transmission in transgenic mice derived from viral-mediated gene transfer [42–45], and so the copy number becomes an important determinant of the expression of the transgene. This is a particularly important consideration in transposon-derived transgenic cattle, given their potential use as bioreactor models. Of the cattle derived from germline-transmission of the transgene, SNU-F1–1 and SNU-F1–2 have three and six copies of integrated transgenes, respectively; this allowed an indirect analysis of the relationship between copy number and expression levels in these animals. Although only two samples, we note that the expression level of the transgene in SNU-F1–2 was almost 2.2-fold higher than in SNU-F1–1, indicating that in this case transposon-mediated integration was not affected by silencing or mosaicism.

Our analysis of transgene expression levels in SNU-F1–1 and SNU-F1–2 suggest that the sites of integration in these animals are valuable positions for targeted genome engineering, as we have not observed effects relating to neighboring gene expression or silencing. Therefore, the transgene integration sites identified in SNU-F1–1 will be used for targeted gene expression studies of site-specific knock-in (KI) transgenesis using the CRISPR/Cas9 system. As a proof of principle for this approach, SNU-F1–1 cells were transfected with a single guide RNA targeting GFP, the Cas9 enzyme and a KI donor template that includes RFP and puromycin resistance transgenes between the homology arms. RFP signal was detected in cells following transfection, suggesting effective targeting by CRISPR/Cas9 (Additional file 8: Figure S6). In addition, we will also investigate whether the transgenes of the F1 generation will again be transmitted to the next generation (F2) and whether the integration sites are altered or remain stable for future generations.

A further issue that we have attempted to address in this study is whether long-term, stable expression of the fluorescent protein in our transgenic cattle (SNU-SB-1, SNU-PB-1, and SNU-F1–1) causes health problems, as GFP has the potential for immunogenicity and cytotoxicity [46]. Female SNU-SB-1, male SNU-PB-1 and F1 calf SNU-F1–1 (male) have all reached maturity at ages of 50, 43 and 19 months old, respectively, and have no general health problems (feeding, growth, body weight, urination or defecation). As further evidence of their health there were no significant abnormalities detected by regular blood analysis (Fig. 2, Additional file 1). These results indicate that transposon-mediated multi-gene integration into the bovine genome and continuous transgene expression does not adversely affect health, for example in organ growth or function. Some RBC, hemoglobin, and hematocrit values in SNU-F1–1 were outside of the reference range (Fig. 2). As previously noted [47], this is most likely due to the age at which blood was collected from the calf, namely at 2 and 10 weeks old. As the genetic variants in SNU-SB-1 and SNU-PB-1 are not significantly different from wild-type cattle, as shown in a previous NGS analysis [5], and the genomic stability (SNP, INDEL and telomere length, etc.) of SNU-F1–1 was not significantly affected (Fig. 5), it is perhaps unsurprising that the cattle generated are healthy. We will continue to monitor their longevity and health status. To our knowledge, this is the first report of transposon-derived transgenic cattle surviving for this length of time without any health issues.

## Conclusions

In this study we have demonstrated that multi-copy transgenic cattle derived using the SB and PB systems have survived for more than 3 years without any health issues, and we show for the first time that their transgenes were stably transmitted through the germline to the next generation. The transgenic calf derived through germline transmission has reached maturity at over 19 months old and is healthy, with no significant abnormalities detected by analyses of blood parameters and genomic stability. In conclusion, our study provides valuable data about the safety and long-term expression of transgenes in cattle using transposon-mediated gene modification, and its utility in applications such as exogenous protein expression.

## Additional files

Additional file 1: Raw data of blood analysis from transgenic cattle. (XLSX 15 kb)

Additional file 2: **Figure S1.** SNU-F1–1 lacks the YFP transgene, as demonstrated by PCR analysis of genomic DNA from SNU-F1–1. PCR was performed using YFP-specific primers. P/C, positive control (SB-CA-YFP vector); WT, genomic DNA from wild type cattle; Tg:SNU-SB-1, genomic DNA from the blood of SNU-SB-1; Tg:SNU-F1–1, genomic DNA from the blood of SNU-F1–1; N/C, negative control (nuclease-free water). (PNG 99 kb)

Additional file 3: **Figure S2.** Detection of the expression of YFP in milk from SNU-SB-1 by confocal microscopy. Images of milk from wild type cattle (left) and SNU-SB-1 (right) taken using a high-throughput confocal microscope. YFP: YFP field. (PNG 1685 kb)

Additional file 4: **Figure S3.** 5′ junction sequence analysis of all integration sites in SNU-F1–1. Sequences showing the genome-to-transposon junctions in the genome of SNU-F1–1 and the integration of transgenes at TTAA sites. (PNG 465 kb)

Additional file 5: **Figure S4.** Germline transmission of GFP expression in uterus, ovary and oocytes from SNU-PB-2. Fluorescent microscope images of GFP expression in: a) uterus from SNU-PB-2, b) ovaries (WT, left; SNU-PB-2, right and arrow) and c) oocytes and cumulus cells from SNU-PB-2. (PNG 1557 kb)

Additional file 6: **Table S1.** All transgene integration sites in SNU-F1–2. (DOCX 24 kb)

Additional file 7: **Figure S5.** Overview of genomic variation in SNU-F1–2. Reference chromosomes from bt1 to btX are denoted by colored boxes at the outer edge. Plots denoting copy number variation (CNV; black dot plots in the green area), coverage (green line plot in the green area) and SNP density (orange histogram in orange area) for the SNU-F1–2 genome are shown for each 10 kb window. (PNG 1244 kb)

Additional file 8: **Figure S6.** CRISPR/Cas9-mediated KI in SNU-F1–1 cells. (a) Schematic of CRISPR/Cas9-mediated KI of the donor construct. (b) SNU-F1–1 cells were co-transfected with the donor plasmid, Cas9 and sgRNA targeting GFP. The detection of RFP signal and loss of GFP signal in these cells suggests that CRISPR/Cas9-mediated homology directed repair has occurred. pCAG: CAGGS promoter; HA: homology arm; BF: brightfield; GFP: GFP field; RFP: RFP field. (PNG 491 kb)

## Abbreviations

ALT: Alanine transaminase
AST: Aspartate transaminase
BUN: Blood urea nitrogen
CNV: Copy number variation
COC: Cumulus–oocyte complex
GFP: Green fluorescent protein
INDEL: Insertion and deletion
IVF: In vitro fertilization
LTR: Long terminal repeat region
NGS: Next-generation sequencing
PB: PiggyBac
RBC: Red blood cells
RFP: Red fluorescent protein
SB: Sleeping Beauty
SNV: Single nucleotide variant
SV: Structural variation
WBC: White blood cells
YFP: Yellow fluorescent protein
