# Supplementary figures and images for: Long-term health and germline transmission in transgenic cattle following transposon-mediated gene transfer
Source: BMC Genomics. 2018 May 23;19:387. doi: 10.1186/s12864-018-4760-4 (PMC5966871; doi:10.1186/s12864-018-4760-4)

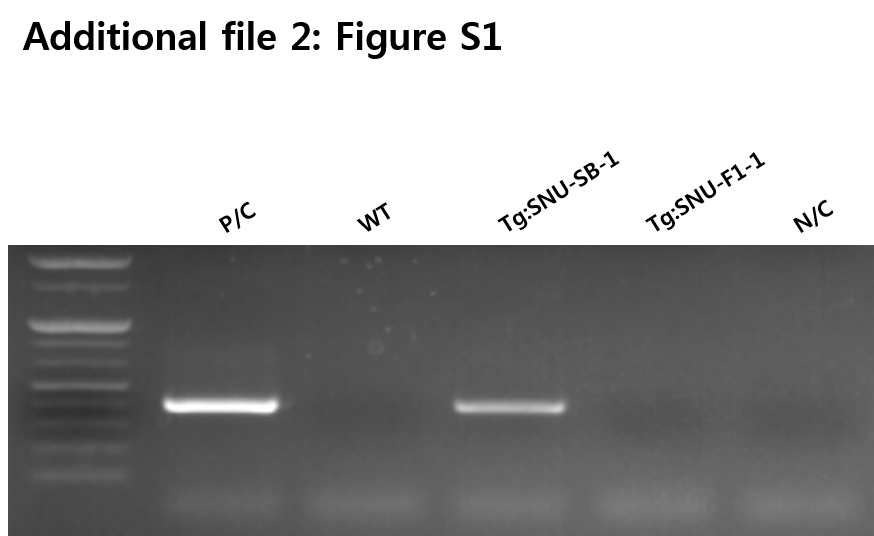

Supplement: Supplementary file 2 — Figure S1. SNU-F1–1 lacks the YFP transgene, as demonstrated by PCR analysis of genomic DNA from SNU-F1–1. PCR was performed using YFP-specific primers. P/C, positive control (SB-CA-YFP vector); WT, genomic DNA from wild type cattle; Tg:SNU-SB-1, genomic DNA from the blood of SNU-SB-1; Tg:SNU-F1–1, genomic DNA from the blood of SNU-F1–1; N/C, negative control (nuclease-free water). (PNG 99 kb) [file 12864_2018_4760_MOESM2_ESM.png]

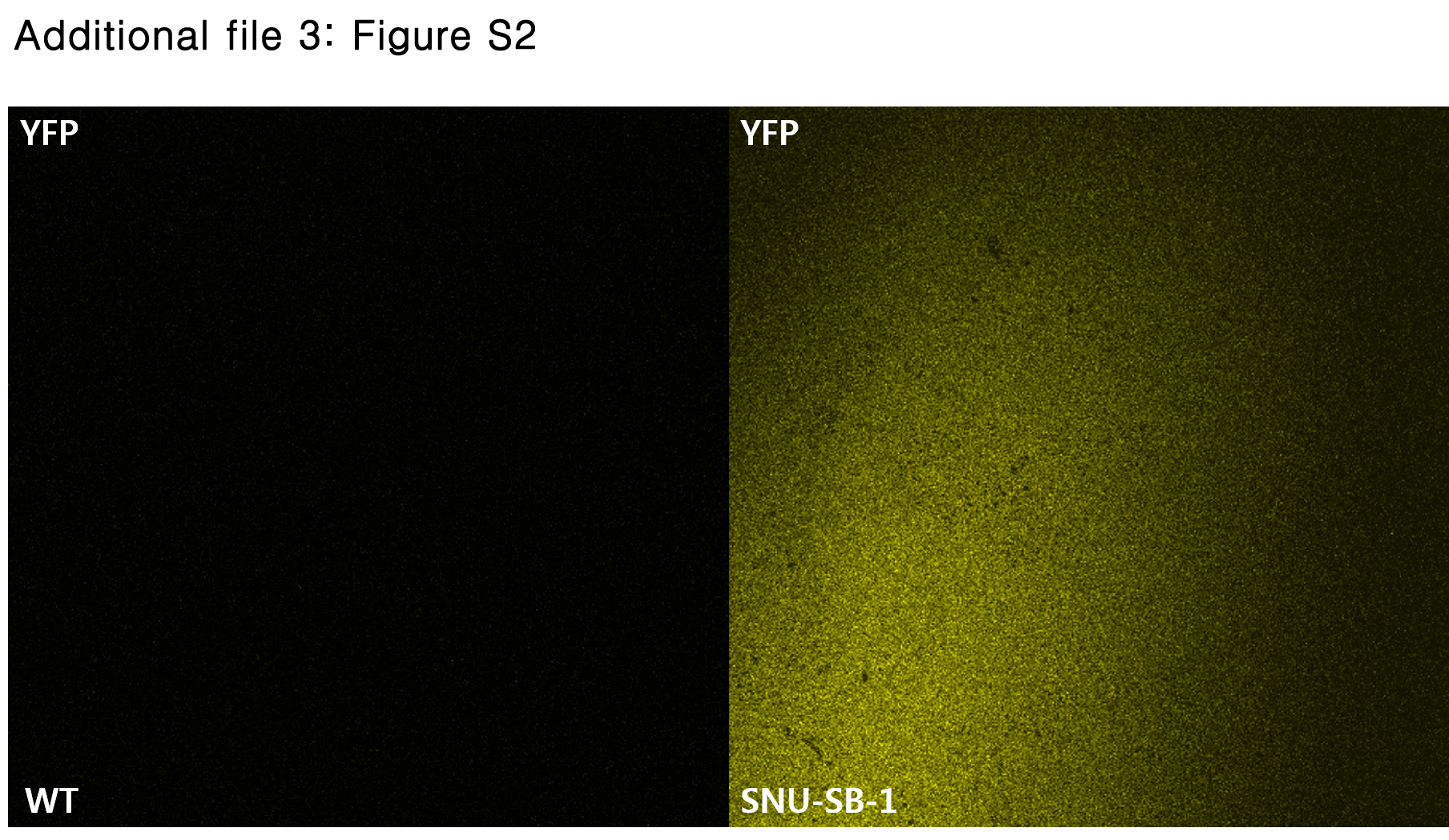

Supplement: Supplementary file 3 — Figure S2. Detection of the expression of YFP in milk from SNU-SB-1 by confocal microscopy. Images of milk from wild type cattle (left) and SNU-SB-1 (right) taken using a high-throughput confocal microscope. YFP: YFP field. (PNG 1685 kb) [file 12864_2018_4760_MOESM3_ESM.png]

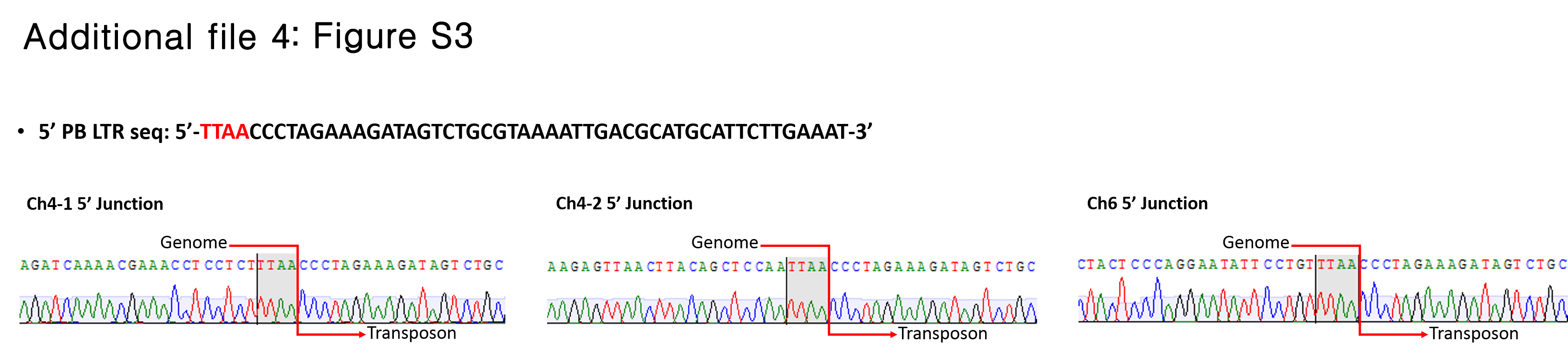

Supplement: Supplementary file 4 — Figure S3. 5′ junction sequence analysis of all integration sites in SNU-F1–1. Sequences showing the genome-to-transposon junctions in the genome of SNU-F1–1 and the integration of transgenes at TTAA sites. (PNG 465 kb) [file 12864_2018_4760_MOESM4_ESM.png]

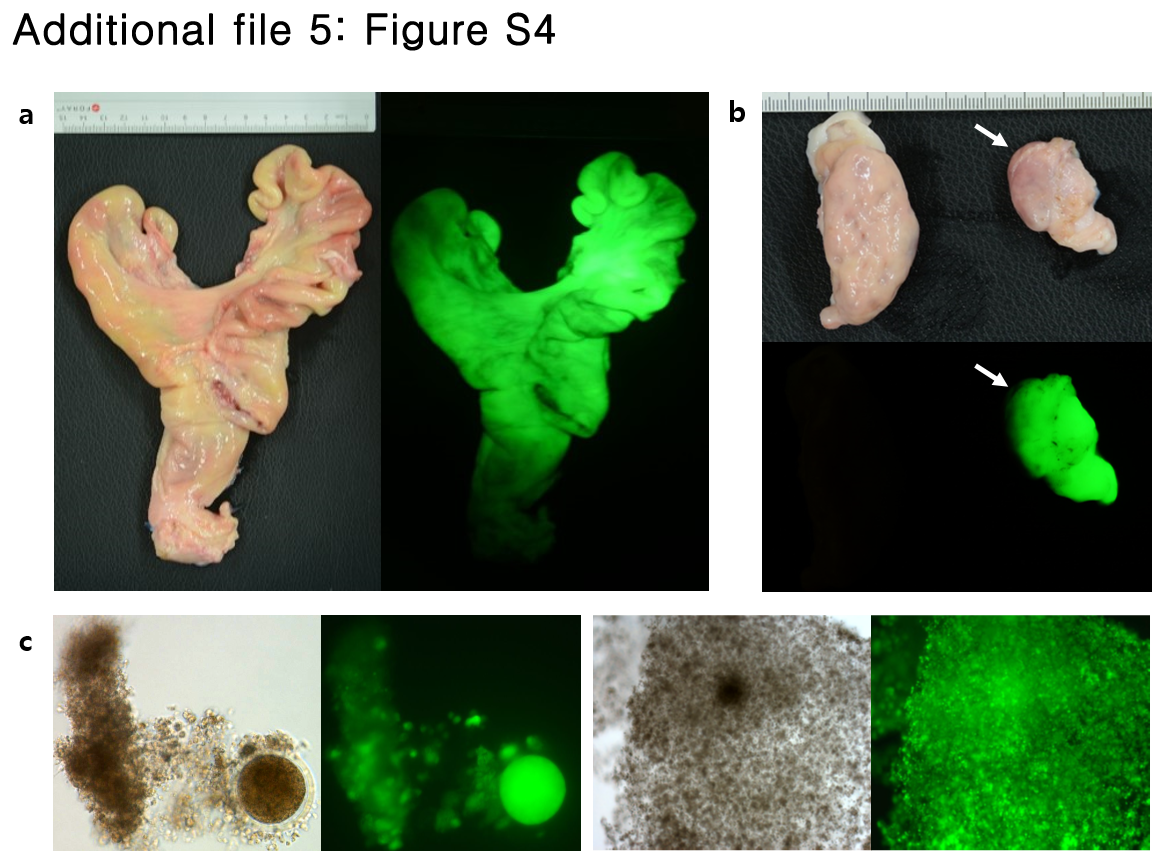

Supplement: Supplementary file 5 — Figure S4. Germline transmission of GFP expression in uterus, ovary and oocytes from SNU-PB-2. Fluorescent microscope images of GFP expression in: a) uterus from SNU-PB-2, b) ovaries (WT, left; SNU-PB-2, right and arrow) and c) oocytes and cumulus cells from SNU-PB-2. (PNG 1557 kb) [file 12864_2018_4760_MOESM5_ESM.png]

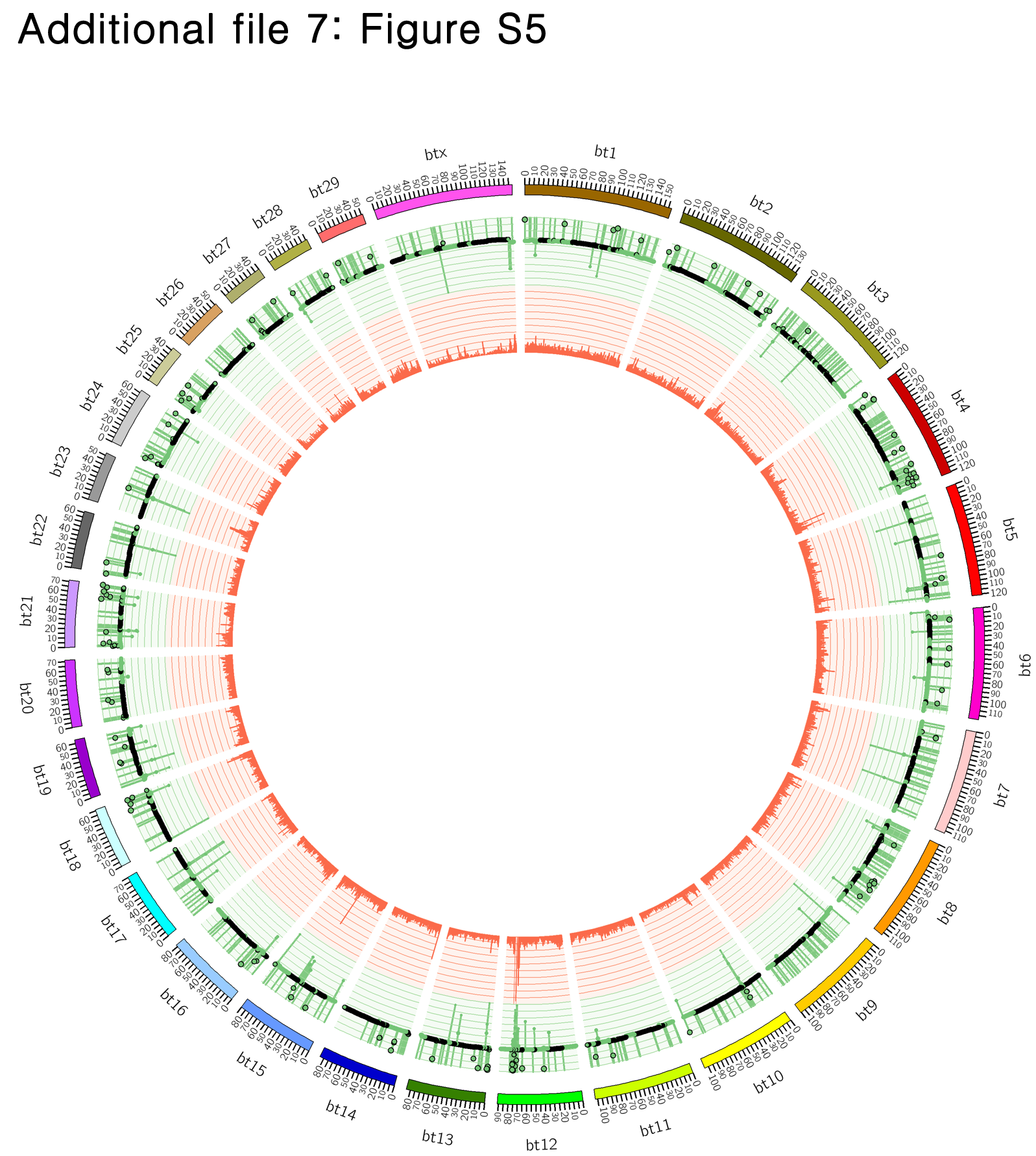

Supplement: Supplementary file 7 — Figure S5. Overview of genomic variation in SNU-F1–2. Reference chromosomes from bt1 to btX are denoted by colored boxes at the outer edge. Plots denoting copy number variation (CNV; black dot plots in the green area), coverage (green line plot in the green area) and SNP density (orange histogram in orange area) for the SNU-F1–2 genome are shown for each 10 kb window. (PNG 1244 kb) [file 12864_2018_4760_MOESM7_ESM.png]

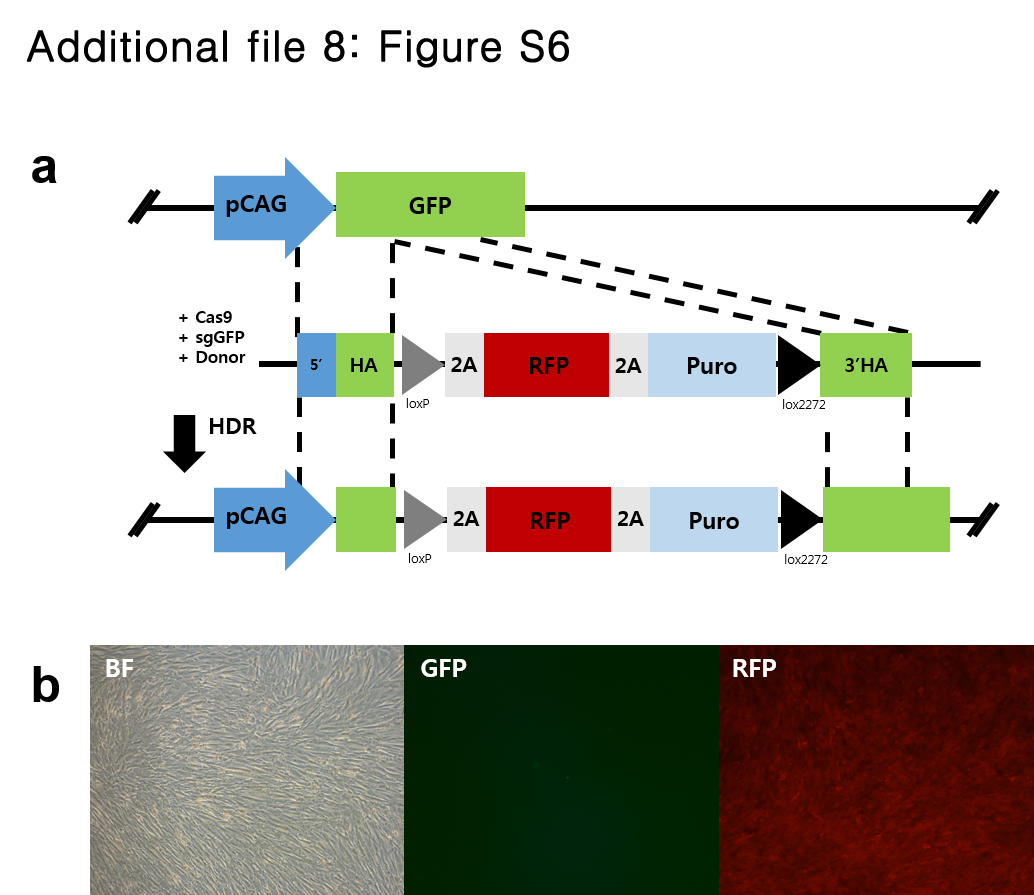

Supplement: Supplementary file 8 — Figure S6. CRISPR/Cas9-mediated KI in SNU-F1–1 cells. (a) Schematic of CRISPR/Cas9-mediated KI of the donor construct. (b) SNU-F1–1 cells were co-transfected with the donor plasmid, Cas9 and sgRNA targeting GFP. The detection of RFP signal and loss of GFP signal in these cells suggests that CRISPR/Cas9-mediated homology directed repair has occurred. pCAG: CAGGS promoter; HA: homology arm; BF: brightfield; GFP: GFP field; RFP: RFP field. (PNG 491 kb) [file 12864_2018_4760_MOESM8_ESM.png]
