# Supplementary material for: Long-term health and germline transmission in transgenic cattle following transposon-mediated gene transfer
Source: BMC Genomics. 2018 May 23;19:387. doi: 10.1186/s12864-018-4760-4 (PMC5966871; doi:10.1186/s12864-018-4760-4)
Supplement: Supplementary file 6 — Table S1. All transgene integration sites in SNU-F1–2. (DOCX 24 kb) [file 12864_2018_4760_MOESM6_ESM.docx]

**Table S1. All integration sites in SNU-F1-2**

| No. | Chromosome | Insertion site | Orientation | Overlapping gene | Location | 5’ gene | 3’ gene |
| --- | --- | --- | --- | --- | --- | --- | --- |
| 1 | 1 | 105665894 | Forward | - | - | ENSBTAG00000025847.3 | ENSBTAG00000011051.5 |
| 2 | 3 | 79750136 | Forward | ENSBTAG00000030852.3 | E2-3 intron | PDE4B | LEPR |
| 3 | 4 | 71122343 | Reverse | - | - | NPVF | C7orf31 |
| 4 | 10 | 85854536 | Forward | LIN52 | E5-6 intron | ALDH6A1 | VSX2 |
| 5 | 12 | 51221667 | Reverse | LMO7 | E14-15 intron | ENSBTAG00000010680.5 | U2 |
| 6 | X | 80581377 | Forward | - | - | PBDC1 | MAGEE2 |
